# Passive Coping Associations With Self-Esteem and Health-Related Quality of Life in Youth With Inflammatory Bowel Disease

**DOI:** 10.3389/fpsyg.2021.670902

**Published:** 2021-06-24

**Authors:** Bonney Reed, Kelly E. Rea, Robyn Lewis Claar, Miranda A. L. van Tilburg, Rona L. Levy

**Affiliations:** ^1^Department of Pediatrics, Emory & Children’s Pediatric Institute, Atlanta, GA, United States; ^2^Department of Psychology, University of Georgia, Athens, GA, United States; ^3^Department of Gastroenterology and Hepatology, University of North Carolina School of Medicine, Chapel Hill, NC, United States; ^4^College of Pharmacy & Health Sciences, Campbell University, Buies Creek, NC, United States; ^5^School of Social Work, University of Washington, Seattle, WA, United States

**Keywords:** coping, quality of life, inflammatory bowel disease, self-esteem, pediatrics

## Abstract

Pediatric patients with inflammatory bowel disease (IBD) may experience chronic stress related to disease symptoms and treatment, with negative consequences to their health-related quality of life (HRQOL). Lower HRQOL among pediatric patients with IBD has been associated with worse disease-related symptoms and psychological functioning, while higher HRQOL has been associated with more adaptive coping with disease symptoms and treatment. In addition, patients’ self-esteem may impact the selection and use of coping strategies through global cognitions about their abilities and perceived competence. The current study seeks to extend existing research on HRQOL in youth with IBD by examining cross-sectional associations among self-esteem and passive coping strategies. Youth ages 9–18 with IBD (*n* = 147) rated their HRQOL using a disease-specific measure, typical strategies used to cope with pain or GI symptoms, and their general self-esteem. Mediation analyses were performed using regression-based techniques and bootstrapping. Results indicated that greater self-esteem was positively associated with HRQOL but negatively associated with passive coping. Controlling for disease activity, age, and gender, significant indirect effects were found in the relation between self-esteem and HRQOL through passive coping. Multiple mediation analyses using the three passive coping subscales found that self-esteem was indirectly associated with HRQOL through its effects specifically on catastrophizing as a passive coping strategy. Results suggest that pediatric patients’ general self-esteem can impact their HRQOL through passive coping and specifically, maladaptive cognitions (e.g., catastrophizing). Interventions aimed at addressing both self-esteem and catastrophizing as a passive coping strategy may offer promise for improving HRQOL in youth with IBD.

## Introduction

Youth diagnosed with inflammatory bowel disease (IBD) may experience significant and chronic stress related to the unpredictable, painful, and potentially embarrassing symptoms associated with an IBD diagnosis. As such, psychological and behavioral functioning among youth with IBD may be impacted in a variety of ways compared to healthy peers, including reduced health-related quality of life (HRQOL; Greenley et al., 2010). HRQOL encompasses an individual’s subjective perception of their physical, mental, and social functioning in relation to their health (Center for Disease Control and Prevention, 2018). Within the pediatric IBD population, lower HRQOL has been associated with increased disease activity, increased pain perception, and greater depressive symptoms (Herzer et al., 2011; Claar et al., 2017). Given the array of challenges faced by youth with IBD and associated relations to poorer HRQOL, there is a great need to understand and promote effective coping with the disease and health-related distress within this population.

Coping refers to efforts to manage stress in the face of adverse experiences, including pain or disease-related distress. The disability-stress-coping model (Wallander and Varni, 1998) theorizes that a youth’s adjustment or distress related to the disease is impacted by both risk and resilience factors. Risk factors include disease activity or symptoms, functional disability related to disease, and psychosocial stressors, while resilience factors include available family or social supports and the use of effective coping strategies. Within this model, coping is theorized to mediate the relations between psychosocial stress and adjustment to medical conditions. Notably, not all cognitive or behavioral coping strategies are adaptive. Adaptive coping strategies, such as active problem solving or social support seeking, are distinct from maladaptive coping, including pain catastrophizing, avoidance, or behavioral disengagement. Adaptive coping strategies are thought to be more organized, flexible, and problem-focused and thus have a positive impact on patient long-term functioning while maladaptive strategies are more disorganized and rigid and are frequently characterized by greater perceived helplessness, thus having a more negative impact on long-term functioning (Skinner et al., 2003). Importantly, maladaptive coping strategies may impede the development of other more adaptive coping resources through negative appraisal of self or situation (Skinner et al., 2003). As such, it is critical to understand and assess coping strategies, particularly among vulnerable populations, including children and adolescents with IBD.

The extent to which patients with IBD engage in maladaptive coping strategies may have important implications for their quality of life, and there is some evidence that youth with IBD may use maladaptive coping strategies more frequently than their healthy peers. For example, compared to healthy peers, youth with IBD have reported greater use of avoidance as a maladaptive behavioral coping strategy (Van der Zaag-Loonen et al., 2004). Notably, pain catastrophizing, a maladaptive cognitive coping strategy, demonstrated the strongest impact on HRQOL among youth with IBD, even when compared to assessments of disease severity and psychological distress (De Carlo et al., 2019). In contrast, among youth using adaptive cognitive coping strategies, including those with greater optimism and perceived control, greater social functioning was reported, while those reporting less perceived control reported poorer social and emotional functioning (Van der Zaag-Loonen et al., 2004). These findings underscore the impact of coping strategies on HRQOL outcomes within the pediatric IBD population. However, other aspects of youth functioning may influence coping; thus, it is not yet clear how to best promote the use of adaptive cognitive and behavioral coping strategies to improve HRQOL among these youth.

Self-esteem is an important perception of the self, varying between individuals, which may relate to the extent to which youth engage in adaptive coping. Self-esteem includes one’s perceived competence and worth regarding their abilities, skills, and qualities, further motivating one’s cognitions and behaviors (Mruk, 2013). Self-esteem is particularly relevant to the coping literature, as the selection and use of either adaptive or maladaptive coping strategies may vary based on cognitions or behaviors influenced by self-esteem. Self-esteem may be further impacted by self-perceptions of appearance, social acceptance, and perceived competence in managing stress, elements which may be uniquely impacted by IBD symptoms and disease course. Interestingly, a recent meta-analysis founds no significant difference in self-esteem between healthy youth and youth with IBD, or youth with IBD compared to youth with other chronic illnesses (Greenley et al., 2010). However, greater disease severity was significantly related to lower self-esteem among adolescents with IBD (Lindfred et al., 2008), suggesting self-esteem may have disease-specific relevance for youth with IBD. Self-esteem has been shown to relate to multiple important outcomes among youth with IBD, including emotional functioning, psychological adjustment, and HRQOL (De Boer et al., 2005). However, the mechanism through which self-esteem relates to these outcomes among youth with IBD is not yet known, nor is it clear how to effectively intervene and improve HRQOL in these youth.

One such possibility is that improved self-esteem may relate to more effective and active cognitive and behavioral coping mechanisms and thus promote better HRQOL, while poorer self-esteem is associated with more maladaptive coping mechanisms and thus poorer HRQOL. Among adults with IBD, greater self-esteem has been associated with greater perceived self-efficacy in coping with life demands (Opheim et al., 2020). As such, self-esteem may impact the differential use of adaptive or maladaptive coping mechanisms in response to disease symptoms and treatment. However, no such studies have examined relations between self-esteem and coping among youth with IBD. Further, an examination of the relations among self-esteem, coping strategies, and resulting impacts on HRQOL appears particularly warranted to further inform interventions aimed at improving HRQOL among youth with IBD. The current study sought to extend existing research on HRQOL in youth with IBD by examining associations between self-esteem and specific maladaptive, passive coping mechanisms as they relate to perceived HRQOL. We hypothesized that the relation between self-esteem and HRQOL among youth with IBD would be mediated by youth’s reported use of passive coping strategies, such that poorer self-esteem would relate to poorer HRQOL through greater use of maladaptive, passive coping mechanisms.

## Materials and Methods

### Participants

The sample consisted of 147 children and adolescents diagnosed with IBD and their parents. Participants were enrolled in a randomized controlled trial (RCT) of a cognitive-behavioral intervention for pediatric IBD patients (Levy et al., 2016). The present paper examines a distinct set of research questions using cross-sectional data collected at baseline prior to randomization or intervention. Interested readers can refer to our previously published papers examining disease and psychosocial characteristics in these participants at baseline (Langer et al., 2014; Van Tilburg et al., 2015; Reed-Knight et al., 2018). Of the 210 dyads enrolled in the RCT, 63 were excluded from the current analysis subsample for the following reasons: baseline assessment not complete (20 dyads), child data missing for assessments included in the current investigation (38 children), and non-independence among siblings as the family had two children with IBD enrolled in the RCT (5 dyads).

Participants were recruited from the gastroenterology departments of Seattle Children’s Hospital in Seattle, WA, and Mary Bridge Children’s Hospital in Tacoma, WA. All procedures were approved by the Institutional Review Boards of both institutions. Inclusion criteria consisted of (1) child age of 8–18 years, (2) child diagnosed with IBD for at least 3 months, (3) child medically approved to engage in normal daily activities at the time of recruitment, (4) child and parent participant had cohabitated for at least the past 3 months, and (5) child and parent English fluency. Exclusion criteria included (1) child diagnosed with a chronic disease other than IBD and (2) developmental disability requiring full-time special education or impairing the ability to participate in study procedures.

### Measures

#### Child-Reported Measures

Children and adolescents completed a battery of questionnaires *via* telephone for the baseline assessment, administered by a nurse researcher. To facilitate comprehension, answer choices were mailed to participants in advance of the telephone session.

##### Self-Esteem

Children’s self-esteem was assessed using the Global Self-Worth subscale from The Self-Perception Profile for Children (Harter, 2012). The Global Self-Worth subscale, conceptualized as analogous to overall self-esteem by Harter, assesses a respondent’s general sense of worth as a person. The subscale evaluates how much one likes oneself as a person, is happy with the way one is leading one’s life, and is generally happy with the way one is, as a human being. Each of the six items on the Global Self-Worth subscale asks respondents to pick which of two descriptions of a person he or she is most like and rate whether the chosen description is “Really True for Me” or “Sort of True for Me.” Each item is then scored on a four-point scale from 1 to 4, where a score of 1 indicates the lowest perceived competence and a score of 4 reflects the highest level of competence. The mean rating is then calculated, with higher scores in the range of 1–4 representing higher perceived self-esteem. The internal consistency for the Global Self-Worth subscale in the current sample was good (*α* = 0.82).

##### Passive Coping

Children’s coping with abdominal pain or other GI symptoms was measured with the Pain Response Inventory (PRI; Walker et al., 1997). The PRI consists of three higher-order scales (active, passive, and accommodative coping). As passive coping has been associated with poorer outcomes in patients with abdominal pain in past research (Walker et al., 2005; Van Tilburg et al., 2015), the current study focused on this domain. The Passive Coping scale consists of Catastrophizing (five items, such as “Think to himself or herself that it’s never going to stop”), Self-Isolation, (five items, such as “Try to be alone.”), and Behavioral Disengagement (five items, such as “Give up trying to feel better”). Parents rated how often their child employed each of these coping strategies on a 0–4 (never to always) scale when experiencing abdominal pain or other GI symptoms. Items on the Passive Coping scale are summed and averaged to obtain a mean subscale score, which can range from 0 to 4.00. The internal consistency for the PRI within the current sample was good (*α* = 0.90).

##### Health-Related Quality of Life

Health-related quality of life was assessed using the 35-item child-report IMPACT-III (Otley et al., 2002). The IMPACT-III is an IBD-specific measure of HRQOL validated in children and adolescents ages 8–17. It is comprised of six domains: bowel symptoms, systemic symptoms, emotional functioning, social functioning, body image, and treatment/interventions. Each item [e.g., *How often did you have to miss out on certain things (hobbies, play, parties) because of your inflammatory bowel disease in the past 2 weeks?*] has five response options, with response labels that differ from item-to-item. To gain a comprehensive representation of children’s HRQOL, the present study utilized the total score, which was calculated by summing all 35 items, with higher scores representing better HRQOL. The possible range for the total HRQOL score is 35–175. In the present study, the internal consistency for the IMPACT-III total score was excellent (*α* = 0.93).

#### Pediatric Gastroenterologist Completed Measures

Each patient’s treating gastroenterologist completed an index of disease activity for study purposes during the patient recruitment visit.

##### Disease Activity

Disease activity was assessed *via* the Pediatric Crohn’s Disease Activity Index (PCDAI; Hyams et al., 1991) for children with Crohn’s disease and *via* the Pediatric Ulcerative Colitis Activity Index (PUCAI; Turner et al., 2007) for children with ulcerative colitis. The PCDAI is a rating of clinical disease activity which incorporates patient report, laboratory values, physician examination results, and growth parameters. PCDAI scores range from 0 to 100. Scores <10 reflect disease remission; scores of 11–30 reflect mild disease activity; and scores >30 reflect moderate to severe disease activity. The PUCAI is a 6-item validated measure of disease activity based on patient reports of symptoms. The PUCAI is completed by pediatric gastroenterologists and scores range from 0 to 85. Scores <10 reflect remission; scores of 10–34 reflect mild disease activity; scores of 35–64 reflect moderate disease activity; and scores of 65 or higher reflect severe disease activity. For the purposes of this study, participants were categorized as experiencing remission/no disease activity or mild/moderate/severe disease activity in Table 1. As has been done in past research, a continuous variable representing disease activity using scores from the PCDAI and the PUCAI was also created in order to control for disease activity in mediation analyses (Reed-Knight et al., 2016). Since the PCDAI and the PUCAI are rated on different scales, PUCAI scores were converted to the PCDAI’s 0 to 100 total scaling by multiplying each PUCAI score by (100/85), resulting in a combined, continuous measure of disease activity for analyses.

**Table 1:** Sample characteristics (*n* = 147 pediatric patients with IBD).

Characteristic	Child
Age, *M* (SD)	13.88 (2.53)
Age, range	9–18
Gender, *n* (%) female	71 (48.3)
Ethnicity, *n* (%) hispanic	6 (4.1)
Race, *n* (%) caucasian	127 (86.4)
Disease, *n* (%)	
Crohn’s disease	101 (68.7)
Ulcerative colitis	46 (31.3)
Disease activity, *n* (%)	
Quiescent	99 (67)
Mild/moderate/severe	48 (33)

#### Analyses

Descriptive statistics and correlation analyses were used to describe the sample in terms of demographics and study variables. Mediation analysis was performed using Hayes’ PROCESS macro, a regression-based path analytic technique (Hayes, 2017). Using an ordinary least squares framework, PROCESS estimates direct and indirect effects in multiple mediator models. To test mediation hypotheses, PROCESS uses bootstrapping to construct confidence intervals for indirect effects through the repeated sampling of the dataset. Findings are based on 5,000 bias-corrected bootstrapped samples. In the event that zero does not lie within the 95% confidence interval for the bootstrapped results for indirect effects, we can conclude that the indirect effect is significantly different from zero and that mediation is demonstrated (Preacher and Hayes, 2004). In the mediation model, disease activity was included as a continuous covariate after computing a combined disease activity score using PCDAI and PUCAI values as described above. Analyses were conducted using IBM Statistical Package for the Social Sciences 26.0.

## Results

### Descriptive Results

Descriptive data for the sample are provided in Table 1. The majority of the sample was classified as having inactive disease based on clinical disease activity indices (67%), and the remaining was classified as experiencing active disease at the time of data collection (33%). Patients classified as experiencing active disease reported significantly lower disease-specific HRQOL (*M* = 122.24, *SD* = 20.51) compared to those classified as experiencing inactive disease (*M* = 135.45, *SD* = 17.22), *t*(145) = 4.24, *p* < 0.001. No differences in self-esteem or passive coping were observed based on disease activity. No differences in self-esteem based on gender were observed, but males reported significantly higher disease-specific HRQOL (*M* = 134.54, *SD* = 18.35) compared to females (*M* = 124.70, *SD* = 20.06), *t*(145) = 3.10, *p* = 0.002. On the Passive Coping subscales, females reported higher levels of self-isolation (*M* = 1.11, *SD* = 1.05 vs. *M* = 0.80, *SD* = 0.87) compared to males, *t* = −1.95, *p* = 0.05. In addition, females reported higher levels of behavioral disengagement (*M* = 0.75, *SD* = 0.86 vs. *M* = 0.48, *SD* = 0.60), *t* = −2.23, *p* = 0.028, and catastrophizing (*M* = 1.12, *SD* = 0.86 vs. *M* = 0.79, *SD* = 0.72), *t* = −2.54, *p* = 0.01, compared to males. Correlations among study variables are provided in Table 2.

**Table 2:** Correlations among study variables and descriptive statistics (*n* = 147).

S.No		1	2	3	4	5	6	7	8	*M* (SD)	Observed range
1	Self-esteem	1.00	−0.42^**^	−0.35^**^	−0.41^**^	−0.30^**^	0.48^**^	−0.15	−0.17^*^	3.29 (0.65)	1.17–4.00
2	PRI: Passive Coping total		1.00	0.82^**^	0.84^**^	0.84^**^	−0.60^**^	0.07	0.28^**^	0.84 (0.70)	0–3.47
3	PRI: Self-Isolation			1.00	0.50^**^	0.47^**^	−0.44^**^	−0.04	0.37^**^	0.95 (0.97)	0–4.00
4	PRI: Behavioral Disengagement				1.00	0.67^**^	−0.52^**^	0.13	0.18^*^	0.61 (0.75)	0–3.80
5	PRI: Catastrophizing					1.00	−0.56^**^	0.11	0.12	0.95 (0.80)	0–3.20
6	HRQOL						1.00	−0.25^**^	−0.38^**^	129.79 (19.76)	68.00–161.00
7	Disease activity							1.00	0.01	10.29 (13.82)	0–76.47
8	Child age								1.00	13.88 (2.53)	9–18

* *p < 0.05 and*

** *p < 0.01*.

*PRI, pain response inventory*.

### Simple Mediation Model

We first examined associations between child-reported self-esteem, disease-specific HRQOL, and passive coping strategies using the Passive Coping scale from the PRI. Disease activity, child age, and child gender were included as covariates given their significant relations with disease-specific HRQOL and passive coping strategies (Table 2). In a simple mediation analysis, self-esteem was indirectly associated with HRQOL through its effect on passive coping. Self-esteem was negatively associated with the use of passive coping strategies (*a* = −0.40), and youth who reported greater use of passive coping strategies reported lower HRQOL (*c* = −11.18). A bias-corrected bootstrap confidence interval for the indirect effect controlling for disease activity, child age, and child gender (*ab* = 4.43) based on 5,000 bootstrap samples was entirely above zero (2.09–7.08).

### Multiple Mediator Model

**Figure 1:**
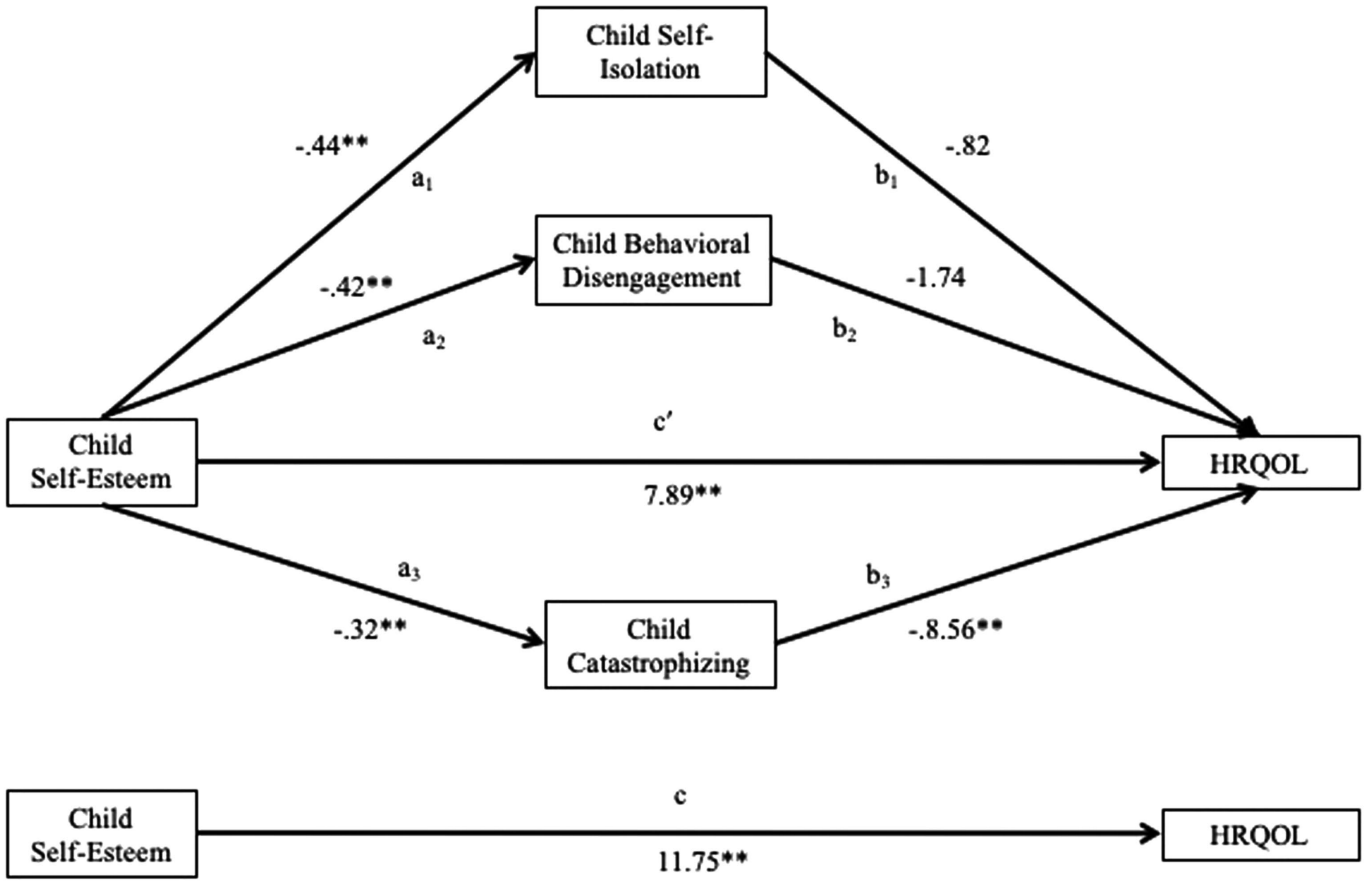
Multiple meditation model of HRQOL. a_3_b_3_, significant indirect effect. Disease activity, child age, and child gender included as covariates. ^*^p < 0.05, ^**^p < 0.01.

As simple mediation analysis supported a relationship between self-esteem and disease-specific HRQOL through the Passive Coping scale, we evaluated a multiple mediator models using the three Passive Coping subscales: Catastrophizing, Behavioral Disengagement, and Self-Isolation. Figure 1 displays the full model with unstandardized B weights for the path coefficients. Multiple mediation analyses conducted using ordinary least squares path analysis found that self-esteem was indirectly associated with HRQOL through its effects specifically on catastrophizing as a passive coping strategy controlling for disease activity, child age, and child gender.

In the full model with the three Passive Coping subscales, self-esteem was negatively associated with self-isolation (a_1_ = −0.44, *p* < 0.01), behavioral disengagement (a_2_ = −0.42, *p* < 0.01), and catastrophizing (a_3_ = −0.32, *p* < 0.01). Only catastrophizing was negatively associated with disease-specific HRQOL (b_3_ = −8.56, *p* < 0.01). Mediated effects were demonstrated by significant indirect effects in the relation between self-esteem and disease-specific HRQOL through catastrophizing [path a_3_b_3_ point estimate = 2.76, standard error (SE) = 1.21, and 95% CI = 0.75 to 5.43]. The full model including mediators and controlling for disease activity, child age, and child gender accounted for 54% of the variance in disease-specific HRQOL; self-esteem alone controlling for disease activity, child age, and child gender accounted for 40% of the variance in disease-specific HRQOL.

## Discussion

Maladaptive coping strategies, including catastrophizing and avoidance, are known to be associated with poorer HRQOL in patients with IBD (De Carlo et al., 2019), though the higher-order factors driving the use of particular coping strategies are less understood. In this study, we sought to investigate relations between global self-esteem, a higher-order construct representing one’s perceived competence and abilities, and disease-specific HRQOL. As expected, higher global self-esteem was associated with higher HRQOL. We hypothesized that global levels of self-esteem may impact the differential use of coping strategies in response to coping with IBD. To evaluate this, we examined passive coping, a maladaptive coping style characterized by catastrophizing, behavioral disengagement, and self-isolation. After finding supports for a model in which global self-esteem is associated with HRQOL through passive coping, we sought to further specify this relationship by examining the three passive coping subscales and interestingly found that catastrophizing alone accounted for the indirect effect in the relation between self-esteem and disease-specific HRQOL.

Results build upon work in adults with IBD, where greater self-esteem has been associated with greater perceived self-efficacy in coping with life demands (Opheim et al., 2020). In the present study, youth with greater global self-esteem were less likely to endorse passive coping through catastrophizing, in which they have negative expectations for their ability to manage pain or symptoms. Results suggest that as youth report higher global perceptions of their competence and abilities they experience lower use of passive coping strategies overall and fewer catastrophic thoughts about their abilities to manage pain or symptoms, both of which were associated with poorer HRQOL. Though not specific to their disease or coping, global self-esteem seems to represent an overarching, higher-order factor impacting the way in which youth think about and cope with the distress of IBD. We controlled for disease activity, child age, and child gender given the relatively large age range of our sample and observed differences in disease-specific HRQOL based on disease activity and child gender, as well as higher levels of passive coping observed in females relative to males. Past research conducted in the Netherlands also founds that male youth with IBD reported higher levels of disease-specific HRQOL compared to females (Van der Zaag-Loonen et al., 2004), and higher levels of passive coping have been observed in females compared to males in non-IBD samples (Hampel and Petermann, 2005). It is well known that girls are more prone to internalizing symptoms from adolescence onwards, and results suggest that passive coping may be at least one risk factor placing girls at increased risk for negative psychological symptoms.

Cognitive-behavioral interventions targeting passive coping and catastrophizing typically address these maladaptive thoughts and behaviors directly, teaching youth to engage in more adaptive ways of thinking about and responding to their pain or symptoms. Such interventions, including work conducted by authors of the current study, have shown strong results in increasing functioning and quality of life for patients with IBD (Levy et al., 2016). However, the present study suggests that addressing global beliefs about overall competence, abilities, and self-worth may offer an additional avenue for improving HRQOL. Such an approach would be more consistent with classic applications of cognitive therapy for depression, in which core beliefs are the ultimate treatment target to change patient’s underlying, maladaptive beliefs about their self-worth (Compton et al., 2004). For a patient with poor global self-esteem, identifying and addressing behavioral deficits in functioning leading to negative self-evaluations, as well as maladaptive thought patterns, may offer an avenue for impacting how he or she approaches to pain and symptom-related thoughts and ultimately improving HRQOL. In addition, research is needed to test whether improvements in functioning and adaptive coping for chronic pain patients treated with exposure-based therapies extend to patients with IBD (Vlaeyen and Crombez, 2019; Lalouni et al., 2020).

Limitations of this study should be considered when interpreting results, which inform future research directions. First, the use of cross-sectional and observational data precludes us from making conclusions regarding causation. Future research should utilize a longitudinal study design to determine how self-esteem and passive coping are temporally related to disease-specific HRQOL and how these factors impact one another over time. Further, empirical exploration of whether interventions aimed at improving self-esteem subsequently decrease the use of passive coping strategies and increase HRQOL is warranted. In the present study, the majority of children was experiencing inactive disease, and results may not generalize to children with active disease. Future research should examine associations between self-esteem, passive coping, and disease-specific HRQOL among children experiencing active disease to determine how disease activity may impact observed relations. Telephone administration of child study questionnaires as opposed to self-administration may have impacted responses if children underestimated symptoms due to social desirability or embarrassment. However, past research has demonstrated comparability between phone interviews and in-person interviews for the assessment of emotional symptoms (Rohde et al., 1997). Additionally, we are unfortunately unable to report data on recruitment rates or potential differences between participants and those who were approached for participation but declined, as these data points were not collected. Finally, the present sample was primarily Caucasian, which may limit generalizability to youth with IBD from different ethnicities. As such, future research is needed to better understand how self-esteem and the use of passive coping strategies are related to HRQOL among youth of different races and ethnicities. While the present model accounted for over half of the variance in disease-specific HRQOL, future research should explore other factors influencing HRQOL and use of specific coping strategies, such as catastrophizing among youth with IBD, including the potential role of family and caregivers’ own coping and the impact of environmental and social contexts on children’s coping with IBD. Such work would be a logical extension of the disability-stress-coping model (Wallander and Varni, 1998) informed by the current study.

This study provides initial evidence using cross-sectional data that global self-esteem, a higher-order construct representing one’s perceived competence and abilities, is associated with the extent to which youth utilize passive coping strategies and engage in catastrophizing, as well as disease-specific HRQOL. Results suggest that a child’s global self-esteem may impact the degree to which adaptive or maladaptive coping strategies are utilized to manage the stressors associated with IBD. As interventions are developed to promote adaptive coping for patients with IBD, it will be important to measure the extent to which beliefs about one’s abilities and competence are associated with uptake of adaptive or maladaptive coping strategies and whether targeting low self-esteem increases utilization of adaptive coping strategies.

## Data Availability Statement

The datasets presented in this article are not readily available because the data continue to be utilized for manuscripts and presentations. Requests to access the datasets should be directed to rlevy@uw.edu.

## Ethics Statement

The studies involving human participants were reviewed and approved by Seattle Children’s Hospital and Mary Bridge Children’s Hospital. Written informed consent to participate in this study was provided by the participants’ legal guardian/next of kin.

## Author Contributions

BR, MT, and RL contributed to the conception and design of the study. BR performed the statistical analysis. BR and KR wrote the first draft of the manuscript. All authors contributed to manuscript revision, read, and approved the submitted version.

### Conflict of Interest

The authors declare that the research was conducted in the absence of any commercial or financial relationships that could be construed as a potential conflict of interest.
